# Postweaning diet affects offspring metabolic outcomes more than maternal diet in male and female mice

**DOI:** 10.14814/phy2.70881

**Published:** 2026-04-20

**Authors:** Adam Corken, Elizabeth C. Wahl, James D. Sikes, Keshari M. Thakali

**Affiliations:** ^1^ Department of Pediatrics University of Arkansas for Medical Sciences Little Rock Arkansas USA; ^2^ Arkansas Children's Research Institute Little Rock Arkansas USA

**Keywords:** high fat diet, indirect calorimetry, maternal programming, metabolism, sex differences, vascular reactivity

## Abstract

The pervasiveness of obesity necessitates expanded pursuits to understand mechanistic drivers of the condition. Maternal diet during conception and gestation can program offspring towards excessive weight gain. Furthermore, the association between obesity and hypertension suggests that maternal programming may impact offspring vascular reactivity. We investigated whether a maternal high fat+sucrose diet (HFD) prior and during pregnancy and lactation affected offspring body composition, metabolic parameters, and vascular reactivity. Using C57BLJ/6 mice, male and female offspring from control (low fat/sucrose) and HFD fed mothers were fed either a control or HFD for 14 weeks after weaning. After 14 weeks of postweaning diets, linear mixed effect modeling adjusting for sex and litter revealed that offspring HFD and sex were significant drivers of body composition changes, non‐fasting serum insulin, serum leptin, and metabolic measures (respiratory exchange ratio, energy expenditure, and food intake). Offspring sex had significant effects on fasting glucose and non‐fasting triglycerides. Neither maternal nor offspring diet or sex had significant effects on mesenteric artery contractility in the presence or absence of perivascular adipose fat. In conclusion, in our experimental paradigm, offspring diet and sex effects on body composition and metabolic parameters overshadowed maternal HFD programming in male and female offspring.

## 1 INTRODUCTION

Currently, four out of every five Americans exhibit an unhealthy bodyweight. Further stratification reveals a majority of these individuals are not just overweight (30.7%) but instead demonstrate exceedingly high body mass index (BMI, kg/m^2^) and high levels of fat mass accumulation denoting an obese (42.4%) and severely obese (9.2%) categorization (Hales et al., 2020). Moreover, obesity is no longer recognized solely as an adult affliction as 19% of all children and adolescents within the US exhibit signs of obesity (Sanyaolu et al., 2019). These figures represent a steady increase observed over several decades with future estimates predicting a continued upward trajectory (Worldwide trends in body‐mass index, 2017). These observations have a clear real‐world impact as countless individuals suffer from co‐morbidities that are either directly related to or exacerbated by obesity thereby requiring treatment or hospitalization which further taxes the already overburdened healthcare system (Malik et al., 2013). Specifically, obesity is associated with increased risk for developing CVD and related disorders such as hypertension (Aronow, 2017; Flora & Nayak, 2019; Garrison et al., 1987; Jiang et al., 2016; Neter et al., 2003; Parker et al., 2016; Srinivasan et al., 1996; Stamler et al., 1978; Wilson et al., 2002; Wühl, 2019). While necessary, these medical interventions to manage the health consequences of obesity results in a $2500–3500 increase in healthcare costs for obese individuals annually in relation to those of normal weight (Cawley et al., 2021). This results in an average of $200–300 billion in additional healthcare expenses annually due to the widening prevalence of obesity (Biener et al., 2017). Thus, further endeavors are warranted to better understand the mechanisms underlying obesity development and progression in order to curtail obesity‐associated health and financial costs.

Conventional wisdom indicates that the largest contributor to excess weight gain is the lifestyle choices of a particular individual such as diet and frequency of physical activity. Research confirms that variations in these characteristics, most notably the consumption of calorie‐dense, nutrient‐poor food, are indeed significant contributors to excessive weight gain (Valicente et al., 2023). However, other studies outline additional factors impacting the propensity for weight gain such as genetics, sleep quality and early life events. Similarly, maternal weight status at conception and throughout gestation has been postulated to predispose offspring to excessive weight gain. This maternal “programming” has been the subject of several investigations which demonstrate that maternal obesity increases offspring's risk of becoming overweight or obese throughout adolescence and adulthood (Godfrey et al., 2017; Tie et al., 2014; Williams et al., 2014). Though these studies have demonstrated a link between maternal habitus during pregnancy and offspring weight gain later on in life, the molecular mechanisms underlying maternal programming and sex differences in offspring outcomes are still under investigation.

In the study described here, we conducted a series of experiments to broaden our understanding of maternal programming's role in the development of accelerated weight gain and vascular perturbations in offspring. We utilized both male and female offspring from mothers fed either a standard chow (control) or high fat diet (HFD) during conception and gestation. We hypothesized that offspring from HFD dams would have increased weight gain, metabolic perturbations, and heightened arterial contractility regardless of postnatal diet when compared to offspring from control dams. The results of the study are as follows:

## 2 MATERIALS AND METHODS

### 2.1 Animals and experimental design

Five‐week‐old female C57BL6/J mice (Jackson Laboratories, Bar Harbor, ME) were given ad libitum access to control diet (17% fat, Inotiv TD.95092) or high fat+sucrose diet (HFD, 45% fat, Inotiv TD.08811) for 12 weeks prior to mating and through pregnancy and lactation. At 17 weeks of age, female mice were bred with lean male mice (fed control diet). Dams and males were housed in opaque (polypropylene) cages, stored on stainless steel racks in a ventilated, climate‐controlled room within the Arkansas Children's Research Institute's animal housing facility. Prior to formation of a mating harem consisting of one male and two dams, females and males were housed grouped by sex with no more than 5 mice per cage. Dam body weight was monitored weekly, and body composition was assessed non‐invasively via QMR (ECHO, Houston, TX, Model # EMR‐035) at 5 and 16 weeks of age. Dam total body mass, fat/lean mass and average litter sizes are available in Figure S1. After birth, litter sizes were adjusted to 6 pups per litter. At weaning (4 weeks of age), offspring were randomized to receive either a control or HFD (same as dam diets mentioned above) ad libitum for 14 weeks, and offspring body weights were monitored weekly. Following weaning, offspring were also housed in matching cage conditions to the breeding harems according to sex in groups no larger than 5 per cage. Body composition of offspring was assessed at weaning (4 weeks of age) and after 14 weeks on respective diets. Percent change in body composition (fat mass or lean mass) was determined by the following formula: (body composition at 18 weeks of age–body composition at 4 weeks)/body composition 4 weeks of age × 100.

After 14 weeks of diets, offspring were euthanized via inhalation of CO_2_ followed by cardiac puncture and exsanguination. Whole blood collected via cardiac puncture was used for serological metabolite analysis. At the time of sacrifice, second order mesenteric arteries (MAs) were also harvested for wire myography contractility assessment as described below. All experimental procedures in mice were approved by the Institutional Animal Care and Use Committee at the University of Arkansas for Medical Sciences.

### 2.2 Indirect calorimetry

After 13 weeks of post‐weaning diets, offspring from control and HFD dams were housed under 12:12 h light–dark cycles in home cages and indirect calorimetry was performed using the Promethion Indirect Calorimetry System (Sable Systems International). Animals were acclimated to the system for 1 week prior to the Promethion measurement period. Data were collected from 3 consecutive 24‐h cycles, though the first 24‐h cycle data was not used for analysis. Mice were allowed unrestricted, ad libitum access to the food hopper and water throughout the study. Respiratory exchange ratio (RER) was calculated as the ratio of carbon dioxide production over oxygen consumption. Energy expenditure (EE) was calculated by using the Weir equation: kcal/h = 60 × [0.003941 × oxygen consumption (VO_2_) + 0.001106 × carbon dioxide production (VCO_2_)].

### 2.3 Body composition, fasting glucose tolerance testing and serum analysis

Offspring body composition was measured via EchoMRI (ECHO, Houston, TX, Model # EMR‐035) before randomization to dietary groups, and at 14 weeks after their respective diets, prior to euthanasia. Glucose tolerance testing was also completed at 14 weeks of diet administration. For glucose testing, mice were fasted for 15 h followed by an intraperitoneal injection of 2.0 g/kg of glucose (PHR1000‐1G, Sigma Aldrich) dissolved in sterile saline. Baseline (0 min) sample collection occurred 2 h before glucose injection. Samples were collected at the following time intervals in relation to glucose administration: 0, 15‐, 30‐, 60‐, and 120‐min post injection. Glucose concentrations were determined using a blood glucose monitor (Bayer Contour Next Meter).

Whole blood collected at euthanasia was used to generate serum, which was then utilized to measure insulin, leptin, and triglyceride (TAG) concentrations for the determination of metabolic characteristics of each group. Serum lipid profiles for triglyceride (TAG) were determined using Fujifilm biochemical colorimetric kits (LabAssay Triglycerides 291‐94501). Insulin and leptin were measured using MSD multi‐array kits (Insulin U‐PLEX K1536HK; leptin U‐PLEX K1535ZK).

### 2.4 Mesenteric artery contractility

Second order MAs were isolated from the mesenteric bed collected at euthanasia. Two mm sections of MAs, either with surrounding perivascular adipose tissue (PVAT) intact or PVAT dissected off, were mounted between two 45 μm wires in a myograph chamber (Danish Myo Technology, 620M). Optimal passive tension was determined using the DMT normalization module that determines the internal circumference at which the vessel would be stretched to the equivalent of a transmural pressure of 100 mmHg. The myograph chamber was filled with warmed (37°C), oxygenated (95% O_2_, 5% CO_2_) physiological salt solution (in mM: NaCl,130; KCl, 4.7; KH_2_PO_4_, 1.18; MgSO_4_ × 7H_2_O, 1.17; CaCl_2_, 1.6; NaHCO_3_, 14.9; dextrose, 5.5; CaNa_2_EDTA, 0.03). A 30‐min equilibration period followed after pulling passive tension and a tissue “wake up” was elicited by administering a bolus of KCl (final concentration of 60 mM) to recapitulate active vessel tension akin to physiological conditions. KCl was then washed out of the sample solution and tissue viability (i.e., contractility/relaxation) was determined with a bolus challenge of 10^−5^ M norepinephrine (NE) or 10^−6^ M Angiotensin II (AngII) to constrict the vessel (DL‐Norepinephrine Hydrochloride, Sigma Aldrich A7256‐1G); (Angiotensin II Acetate Salt Hydrate, Sigma Aldrich A2900‐5MG) followed by 10^−5^ M acetylcholine (ACh) (Acetylcholine Chloride, Sigma Aldrich A9101‐10VL) to facilitate vessel relaxation. The maximum contractile forces for NE and AngII were normalized to the maximum contractile force elicited by the KCl “wake up,” while ACh‐induced relaxation in NE‐preconstricted arteries was normalized relative to NE‐induced constriction. Contractility data are represented at mean ± SEM.

### 2.5 Statistical analysis

Initial statistical analysis was performed by one‐way ANOVA with a Tukey's post hoc comparison to determine statistically significant differences of each measured variable amongst groups of the same sex using GraphPad Prism (version 10.4.1, Boston, MA, USA). For this primary analysis, individual offspring were used as the unit of analysis.

Secondary statistical analyses were performed in R (version 2023.06.2, R Foundation for Statistical Computing, Vienna, Austria) using the lmerTest package. To account for the non‐independence of littermates, all offspring outcomes were analyzed using linear mixed‐effects models with dams included as a random intercept. Fixed effects included maternal diet (Control vs. HFD), offspring postweaning diet (Control vs. HFD), offspring sex (Male vs. Female), and their interaction where applicable. Model estimates were obtained using restricted maximum likelihood (REML). *t*‐tests with Satterthwaite's method were used to calculate approximate degrees of freedom and *p*‐values for fixed effects. For each outcome, the model was specified as follows:

Outcome ~ DamDiet × OffspringDiet + OffspringSex + (1 | Dam)

Outcomes included body composition measures (fat and lean mass changes), metabolic parameters (non‐fasted insulin, fasting glucose, serum leptin, serum TAG, average 24‐h energy expenditure, cumulative 48‐h food intake, and average 24‐h resting energy expenditure), and vascular reactivity (NE, ACh, or NE‐induced responses with or without PVAT). Significance was defined as *p* < 0.05. Post hoc comparisons were conducted as appropriate. Model diagnostics were evaluated using residual plots, and singular fits were noted when random effect variance was estimated near zero. In cases of singular fit, the random effect was retained for consistency but interpreted cautiously.

Sample sizes vary between analyses due to missing measurements or use of subsets of animals for specific assays. For body mass analysis, 42 dams were used to generate offspring for analyses. For body composition (lean mass, fat mass), indirect calorimetry, and food intake, 47 dams were used to generate offspring for analyses. For serum measurements 14–26 dams were used to generate offspring for analyses, and for contractility experiments, 20 dams were used to generate offspring for analyses.

Previously for the collection of mouse mesenteric artery contractility data where SD was 0.15 mN, 10 animals per group will provide about 80% power to detect a mean difference of 0.3 in arterial contractility when performing a two‐tailed *t*‐test, while controlling type I error at 0.05; (March 2022, Piface v1.76, Retrieved January 3, 2022).

## 3 RESULTS

### 3.1 Maternal and postweaning diet effects on offspring bodyweight and body mass composition changes

**FIGURE 1:**
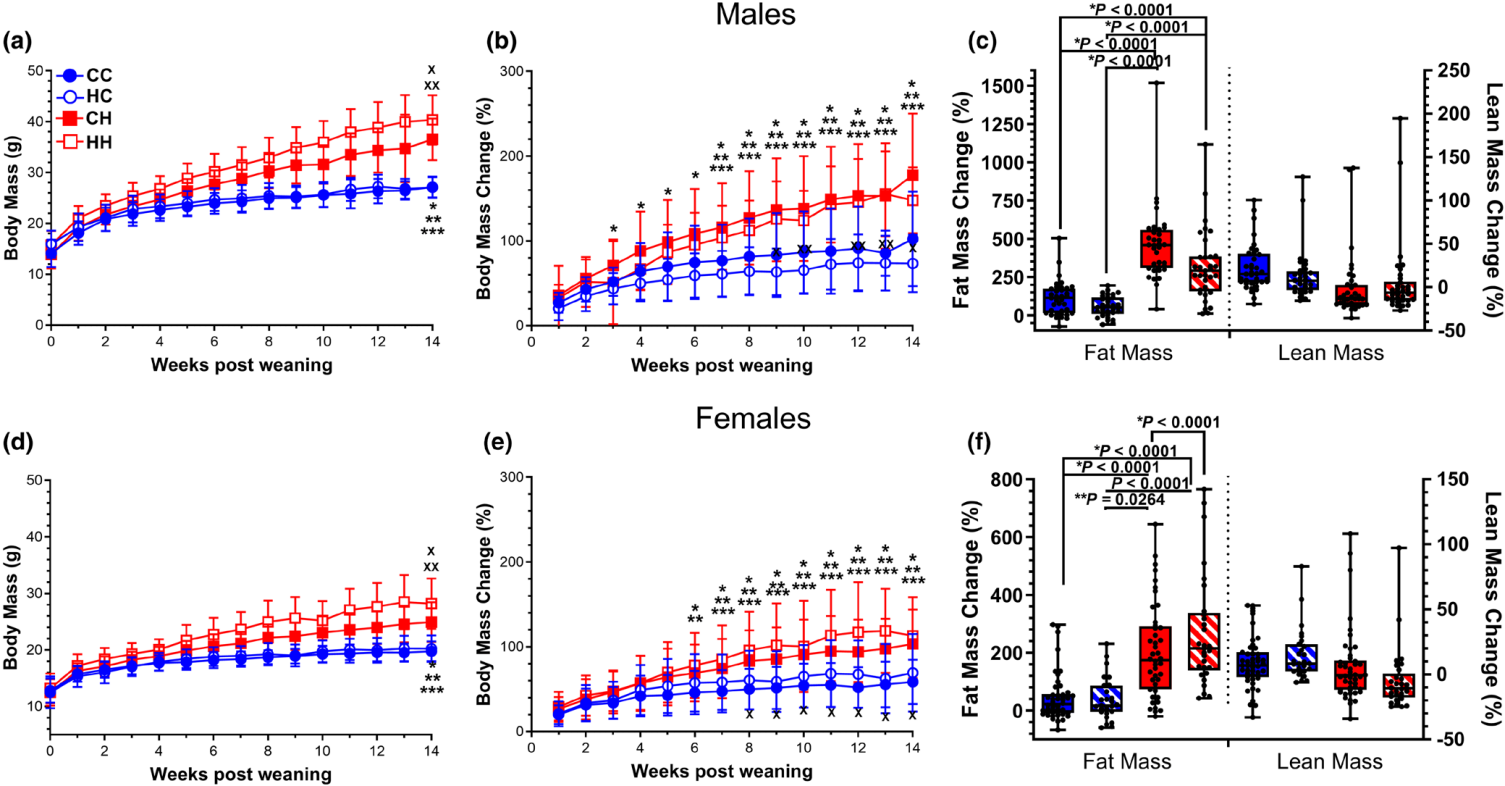
Body mass and composition of male and female offspring. (a) Body mass accumulation for male offspring from weaning to the study's conclusion after 14 weeks of feeding. *p < 0.005 [CC vs. HH]; **p < 0.05 [CC vs. CH]; ***p < 0.005 [HC vs. CH]; ^x^ *p* <0.005 [HC vs. HH]; ^xx^ *p* <0.05 [CH vs. HH]. (b) Percentage total body mass change relative to baseline (weaning) for male offspring. *p < 0.005 [HC vs. CH]; **p < 0.05 [CC vs. CH]; ***p < 0.01 [HC vs. HH]; ^x^ *p* <0.005 [CC vs. HC]; ^xx^ *p* <0.005 [CC vs. HH]. (c) Percentage changes of fat and lean mass after 14 weeks on diets in male offspring. *p* < 0.0001 (Fat mass CC vs. HH, CC vs. CH, HC vs. HH, HC vs. CH). (d) Body mass accumulation for female offspring over 14 weeks on diets. *p < 0.005 [CC vs. CH]; **p < 0.005 [CC vs. HH]; ***p < 0.05 [HC vs. CH]; ^x^ *p* <0.01 [HC vs. HH]; ^xx^ *p* <0.005 [CH vs. HH]. (e) Percent changes in total body mass relative to baseline for female offspring. *p < 0.05 [CC vs. CH]; **p 0.05 [HC vs. CH]; ***p < 0.05 [CC vs. HH]; ^x^ *p* <0.05 [HC vs. HH]. (f) Percentage changes of fat and lean mass after 14 weeks on diets in female offspring. *p* < 0.0001 (Fat mass: CC vs. HH, CC vs. CH, HC vs. HH, CH vs. HH). *p* = 0.0264 (Fat mass: HC vs. CH). Male Offspring: *N* = 42 (CC); *N* = 32 (HC); *N* = 46 (CH); *N* = 36 (HH). Female Offspring: *N* = 56 (CC); *N* = 28 (HC); *N* = 57 (CH); *N* = 30 (HH). Box plots depict the lower and upper quartile of the data sets as the outer borders with median value in between. The whiskers represent the minimum and maximum values of the data set. Circles represent individual mice.

Male and female offspring from both control and HFD fed dams were administered either a control or HFD for 14 weeks after weaning. Over the course of the study, postweaning control diet fed males demonstrated a reduced propensity for weight gain when compared to postweaning HFD fed males (Figure 1a). With regards to weight gain over the 14‐week postweaning diet challenge, initial one‐way ANOVA showed that control diet fed male offspring from control diet mothers (CC) were indistinguishable from the matching control diet fed cohort from HFD dams (HC) while HFD fed offspring from control mothers (CH) did not significantly differ from HFD fed males from HFD dams (HH). The male CC and HC groups differed significantly from CH and HH in terms of overall weight changes from the introduction of the offspring diet (weaning) to the conclusion of the study (Figure 1b) which suggests that in our diet feeding paradigm, offspring diet had a much greater effect on overall weight gain in male offspring than did maternal diet. When analyzing the change in body composition over the 14‐week period, both male offspring HFD groups (CH and HH) had marked increases in fat mass accumulation relative to control fed groups (CC and HC, Figure 1c).

In female offspring, initial one‐way ANOVA analysis of weight accumulation over the 14‐week period followed a similar trend relative to male littermates as the control diet fed groups trended together while the HFD fed groups did so similarly (Figure 1d). Of note, there appeared to be a greater stratification in terms of mass change over the course of the 14‐week period for female offspring as CC females demonstrated the smallest overall change in weight while HH individuals had on average the highest percentage weight change (Figure 1e). The weight gain in these CC females differed not only from the HFD groups (CH and HH) but also from the HC cohort. Similarly, HH females differed not only from control groups (CC and HC) but from the CH cohort as well. This implies that there is a maternal component for females when programming for minimal and maximal overall weight gain. Furthermore, in female offspring, a maternal programming effect was seen with both lean and fat mass changes over the 14‐week offspring feeding challenge (Figure 1f). When assessing fat mass increases, female CC offspring did not differ from female HC offspring, though postweaning control fed groups were markedly different from those fed a postweaning HFD. Female HH offspring on average had the highest accumulation of fat mass even in comparison to CH offspring. Raw lean and fat mass values for male and female offspring are shown in Figure S2.

Secondary linear mixed‐effects modeling analysis confirmed initial ANOVA results that postweaning diet significantly influenced weight gain trajectories (Week × offspring diet, *p* < 0.0001), with additional modulation by sex (Week × offspring sex, *p* < 0.0001). Higher‐order interactions were observed for Week × dam diet × offspring diet (*p* = 0.031) and Week × offspring diet × sex (*p* = 0.0035). No significant main effects of maternal diet or offspring diet were detected (*p* > 0.05). Linear mixed‐effects modeling with dam as a random effect showed a significant effect of offspring HFD on percent change in body mass after 12 weeks on diets (estimate = 55.565%, *p* = 0.000735) and a trend for sex effect (females gained less; estimate = −25.039, *p* = 0.054841). Regarding changes in fat mass percentage after 14 weeks on diets, linear mixed‐effects modeling with dam as a random effect showed a strong effect of offspring HFD on fat mass gain (estimate = 231.670%, *p* < 0.001), a significant sex effect (females gained less; estimate = −114.595%, *p* < 0.001). Likewise, for percent change in lean mass, linear mixed‐effects modeling accounting for dam as a random effect showed that female offspring gained significantly less lean mass than males (estimate = −7.6692%, *p* = 0.010802) and that offspring HFD was associated with significantly decreased lean mass (estimate = −19.3688%, *p* < 0.001).

### 3.2 Postweaning diet impacts serum leptin and triglycerides more than maternal diet

**FIGURE 2:**
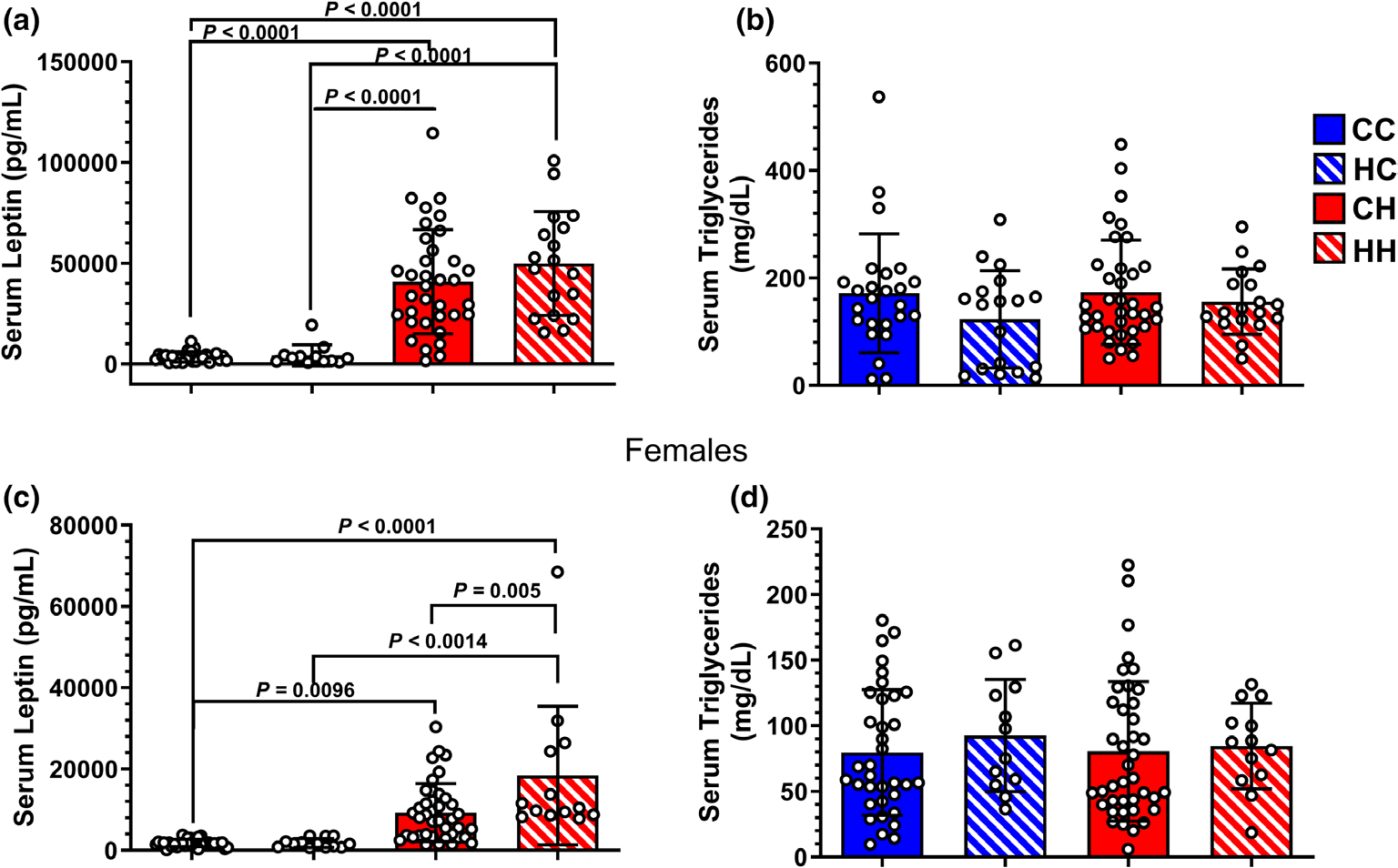
Serological assessment of leptin and triglyceride. (a) Serum leptin and (b) Triglyceride levels for male offspring after 14 weeks of post‐weaning diet administration. *p* < 0.0001 (Leptin: CC vs. HH, CC vs. CH, HC vs. HH, HC vs. CH). (c) Female offspring serum leptin and (d) TAG levels following 14 weeks of post‐weaning diet. *p* < 0.0001 (Leptin: CC vs. HH), *p* = 0.0014 (Leptin, HC vs. HH), *p* = 0.005 (Leptin: CH vs. HH), *p* = 0.0096 (Leptin: CC vs. CH). Male Offspring: *N* = 26 (CC); *N* = 32 (HC); *N* = 18 (CH); *N* = 17 (HH). Female Offspring: *N* = 36 (CC); *N* = 40 (HC); *N* = 13 (CH); *N* = 13 (HH).

For male offspring, the postweaning control diet groups had the lowest leptin levels after 14 weeks on the diet, while those fed a postweaning HFD had markedly elevated serum leptin levels (Figure 2a). Initial one‐way ANOVA results showed that the male HH group was indistinguishable from the CH group. Additionally, we assessed serum triglyceride levels, which likewise correlate with weight and fat mass status (Aristizabal et al., 2016; Haidari et al., 2002; Haluzik et al., 2004). There were no significant differences in serum triglyceride levels between any of the male cohorts after 14 weeks of postweaning diets (Figure 2b).

For the female cohort, like their male counterparts, the postweaning control diet groups had the lowest serum leptin serum levels while the postweaning HFD groups had the highest leptin levels (Figure 2c). Initial one‐way ANOVA results showed that female HH offspring had significantly higher leptin levels than female CH offspring. Like the males, there was no difference between female groups when assessing serum triglyceride levels (Figure 2d).

Secondary linear mixed‐effects modeling accounting for dam as a random effect revealed a significant effect of offspring diet on serum leptin (HFD > control; estimate = 20277.17 pg/mL, *p* < 0.001) and a significant effect of offspring sex on serum leptin (female < male; estimate = −17672.59 pg/mL, *p* < 0.001). Neither maternal diet nor the interaction between maternal and offspring diet significantly affected leptin levels. Linear mixed‐effects modeling accounting for dam as a random effect revealed a significant effect of offspring sex on serum triglycerides (females < males; estimate = −79.909 mg/dL, *p* < 0.001). Neither maternal diet, offspring diet, nor their interaction significantly affected serum triglyceride levels.

### 3.3 Offspring diet predominantly determined non‐fasting serum insulin and fasting glucose clearance

**FIGURE 3:**
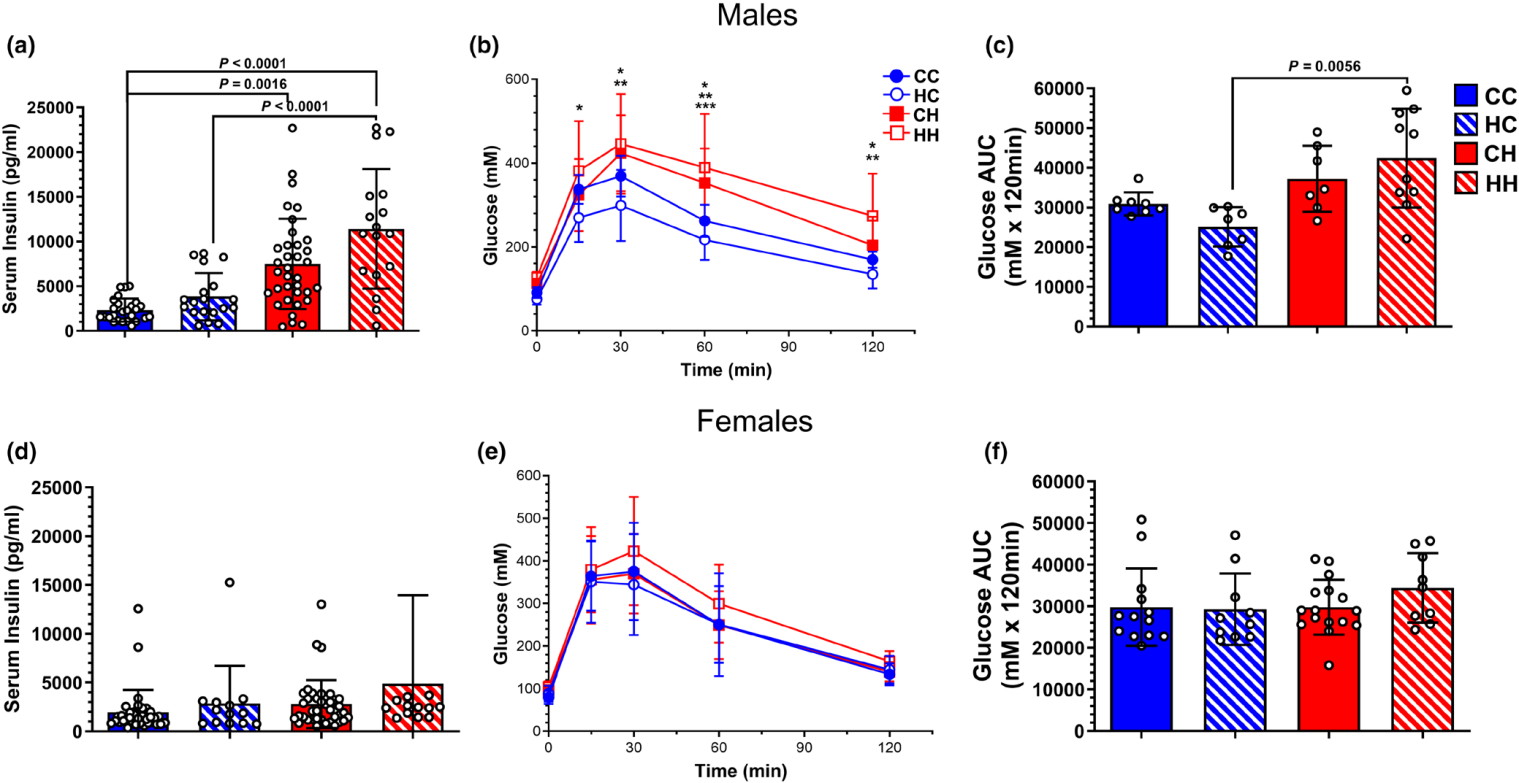
Non‐fasting serum insulin and fasting glucose tolerance profiles. (a) Male offspring non‐fasting serum insulin levels following 14 weeks of post‐weaning dietary administration. *p* < 0.0001 (Insulin: CC vs. HH, HC vs. HH), *p* = 0.0016 (Insulin: CC vs. CH). (b) Fasting glucose tolerance profile for male offspring. *p < 0.05 (Glucose: HC vs. HH), **p < 0.05 (Glucose: HC vs. CH); ***p < 0.05 (Glucose: CC vs. HH). (c) AUC analysis of glucose tolerance test scatterplot profiles. *p* = 0.0056 (HC vs. HH). (d) Female offspring non‐fasting serum insulin level. (e) Female offspring fasting glucose tolerance test scatterplot profile. (f) Glucose tolerance test profile AUC. Male Offspring: *N* = 26 (CC); *N* = 32 (HC); *N* = 18 (CH); *N* = 17 (HH) for serological analysis. Female Offspring: *N* = 36 (CC); *N* = 40 (HC); *N* = 13 (CH); *N* = 13 for serological analysis. Male Offspring: *N* = 8 (CC, HC, CH); *N* = 10 (HH) for glucose tolerance testing. Female Offspring: *N* = 12 (CC); *N* = 11 (HC); *N* = 16 (CH); *N* = 9 (HH) for glucose tolerance testing.

After 14 weeks on respective diets, initial one‐way ANOVA analysis showed a marked increase in non‐fasting serum insulin levels for both postweaning HFD groups when compared to those receiving postweaning control diets in the male offspring (Figure 3a). Though non‐fasting insulin levels in CC and HC males did not differ, the HH group had on average significantly higher non‐fasting insulin levels when compared to CH, thereby suggesting that a maternal HFD may program higher non‐fasting insulin levels in male offspring fed a postweaning HFD. Additionally, fasting blood glucose was assessed at 0, 15, 30, 60, and 120 min following an injection of intraperitoneal glucose (Figure 3b). Interestingly, when comparing the area under the curve (AUC) of the glucose tolerance test (GTT), maternal and offspring diets exhibited markedly different patterns compared to those observed for body composition and serum labs. HH animals exhibited significantly higher fasting glucose levels when compared to both postweaning control diet groups; however, CC and CH cohorts were not different from each other (Figure 3c). Furthermore, HC animals had the lowest blood glucose levels when compared to all other groups.

When we assessed non‐fasting insulin levels in the female population, there was no discernable difference noted amongst cohorts regardless of maternal or offspring diet combination (Figure 3d). Moreover, our assessment of fasting blood glucose levels in female offspring revealed no discernible difference in any given group in comparison to all others (Figure 3e,f).

Secondary linear mixed‐effects modeling accounting for dam as a random effect revealed significant effects of offspring HFD diet (estimate = 2663.3 pg/mL, *p* = 0.0004), offspring sex (females < males, estimate = 3208 pg/mL, *p* < 0.001), and a significant maternal × offspring diet interaction (estimate = 3049.7 pg/mL, *p* < 0.001) on non‐fasting serum insulin. No main effect of maternal diet was observed. There was a trend for an interaction between maternal diet and offspring diet (estimate = 2559.7 pg/mL; *p* = 0.056420). Linear mixed‐effects modeling accounting for dam as a random effect revealed significant effects of offspring sex (females < males, estimate = −5290.40 mM × 120 min, *p* = 0.0162) on fasting glucose clearance (area under the curve). No main effect of postweaning diet was observed.

### 3.4 Offspring diet had greater effects than maternal diet on offspring substrate utilization, energy expenditure, and cumulative food intake

**FIGURE 4:**
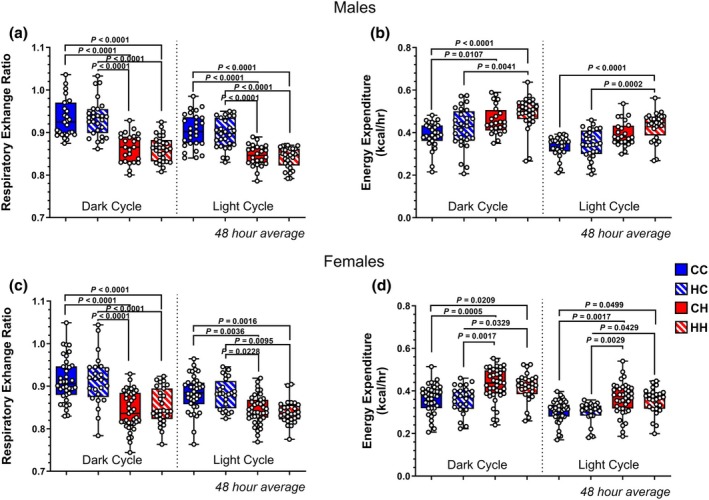
Indirect calorimetry results in male and female offspring. (a) Average respiratory exchange ratios for male offspring over a 48‐h period for both the dark and light cycles. *p* < 0.0001 (CC vs. HH dark and light). (b) Male offspring energy expenditure averaged over a 48‐h period for both light and dark cycles. *p* < 0.0001 (CC vs. HH dark and light), *p* = 0.0107 (CC vs. CH dark), *p* = 0.0041 (HC vs. HH dark), *p* = 0.0002 (HC vs. HH light). (c) Average female offspring average respiratory exchange ratio for both dark and light cycles over 48 h. *p* < 0.0001 (CC vs. HH dark), *p* = 0.0016 (CC vs. HH light), *p* = 0.0036 (CC vs. CH light), *p* = 0.0035 (HC vs. HH light), *p* = 0.0228 (HC vs. CH light). (d) Female offspring energy expenditure averaged a over 48 h period for the light and dark cycles. *p* = 0.0209 (CC vs. HH dark), *p* = 0.0005 (CC vs. CH dark), *p* = 0.0329 (HC vs. HH dark), *p* = 0.0017 (HC vs. HH dark), *p* = 0.0499 (CC vs. HH light), *p* = 0.0017 (CC vs. CH light), *p* = 0.0429 (HC vs. HH light), *p* = 0.0029 (HC vs. CH light). Male Offspring: *N* = 27 (CC); *N* = 27 (HC); *N* = 24 (CH); *N* = 28 (HH). Female Offspring: *N* = 39 (CC); *N* = 24 (HC); *N* = 39 (CH); *N* = 27 (HH). Box plots depict the lower and upper quartile of the data sets as the outer borders with median value in between. The whiskers represent the minimum and maximum values of the data set. Circles represent individual mice.

We next investigated whether maternal diet affected offspring substrate utilization for deriving energy after 14 weeks on respective diets. In initial one‐way ANOVA analysis, in male offspring, the postweaning control diet fed mice had a higher average RER over a 48‐h period (>0.90) for both dark and light cycles indicating that carbohydrates were the primary substrate utilized for energy (Figure 4a). Furthermore, the postweaning HFD offspring groups had lower RERs (~0.80), indicating more fat utilization, providing confirmation of postweaning diet macronutrient composition. Maternal diet had no effect on male offspring RER when comparing offspring on the same postweaning diets (CC vs. HC or CH vs. HH). In male offspring, expenditure (EE) values were averaged over a 48‐h period for both the light and dark cycles, and there were significant differences in EE among groups driven primarily by offspring diet (Figure 4b). There were no differences in cumulative food intake over a 48‐h period in either the light or dark cycles between the groups of male offspring (Figure S3).

In initial one‐way ANOVA analysis, in female offspring the postweaning control fed cohorts trended towards higher average RER values whereas the postweaning HFD group had lower RER, similar to males for both dark and light cycles (Figure 4c) suggesting substrate utilization is largely dependent on offspring diet. In female offspring, HH females had significantly higher EE when compared to postweaning control diet fed offspring for both the light and dark cycles (Figure 4d). In both the light and dark cycles, cumulative food intake over a 48‐h period was greater in CH females compared to postweaning control diet fed female offspring (CC and HC, Figure S3).

Linear mixed‐effects modeling accounting for dam as a random effect revealed significant effects for reduced RER in offspring from HFD dams (estimate = −0.049918, *p* < 0.001) and in female offspring (estimate = −0.013023, *p* = 0.00475). No significant effects of offspring diet or Dam × Offspring diet interaction were observed, indicating that RER is largely unaffected by early‐life dietary exposures. Linear mixed‐effects modeling accounting for dam as a random effect revealed significant effects of offspring HFD diet (estimate = 0.049622 kcal/h, *p* < 0.001) and female sex (estimate = −0.039296 kcal/h, *p* < 0.001) on energy expenditure during both the light and dark cycles. Linear mixed‐effects modeling accounting for dam as a random effect revealed a significant effect of offspring HFD diet (estimate = 5.275 g, *p* < 0.001) on cumulative 48 h food intake (total, light and dark cycle), but no effect of maternal diet, offspring sex, or interaction between maternal and offspring diet on 48‐h cumulative food intake.

### 3.5 Maternal and offspring HFD had minimal impact on mesenteric artery vascular reactivity

**FIGURE 5:**
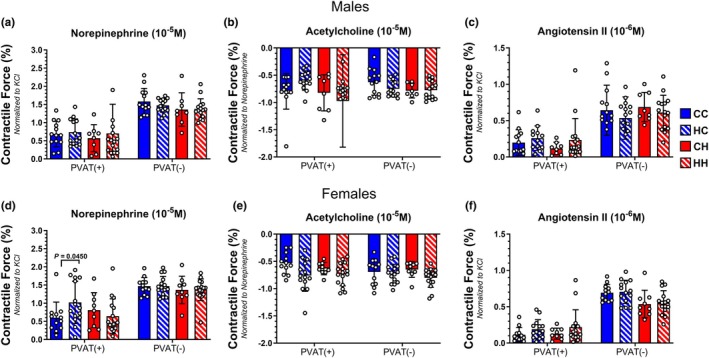
Arterial contractility and relaxation of second order mesenteric arteries, with or without attached perivascular adipose tissue (PVAT), from male and female offspring. (a) Norepinephrine (NE)‐induced contraction in mesenteric arteries from male offspring, with and without PVAT attached. (b) ACh‐induced relaxation in NE‐precontracted mesenteric arteries from male offspring. (c) Angiotensin II (AngII)‐induced contraction in mesenteric arteries from male offspring, with and without PVAT attached. (d) Norepinephrine (NE)‐induced contraction in mesenteric arteries from female offspring, with and without PVAT attached *p*(value) = 0.0450 (CC vs. HC). (e) ACh‐induced relaxation in NE‐precontracted mesenteric arteries from female offspring. (f) Angiotensin II (AngII)‐induced contraction in mesenteric arteries from female offspring, with and without PVAT attached. Male Offspring: *N* = 13 (CC); *N* = 14 (HC); *N* = 8 (CH); *N* = 16 (HH). Female Offspring: *N* = 12 (CC); *N* = 15 (HC); *N* = 9 (CH); *N* = 16 (HH).

Vascular reactivity of 2nd order mesenteric arteries was assessed using the contractile agonists NE and AngII, and ACh was used to assess endothelium‐dependent relaxation. Furthermore, assessment of arterial contractility was conducted in the presence and absence of surrounding PVAT since PVAT can regulate arterial tone via the paracrine release of soluble mediators (Chang et al., 2020). In initial one‐way ANOVA analysis, in male offspring, NE‐induced mesenteric artery contractility was not different between groups, regardless of the presence or absence of PVAT (Figure 5a). Removing PVAT trended to increase NE‐induced contractility forces for all groups. There were no differences in endothelium‐dependent relaxation to ACh between the groups of male offspring, either in the presence of absence of PVAT (Figure 5b). Of note, removal of PVAT did not affect ACh‐induced relaxation for any of the groups of male offspring. There were no differences in arterial contractility to AngII among the groups of male offspring (Figure 5c).

Interestingly, when assessing NE‐induced contraction in female offspring, initial one‐way ANOVA analysis showed that mesenteric female HC offspring had greater contraction to NE relative to the CC cohort when PVAT remained intact (Figure 5d). The absence of PVAT, all groups of female offspring had greater NE‐induced contraction (compared to the PVAT intact mesenteric arteries), and there were no differences between the groups (Figure 5d). Like male offspring, no difference in ACh‐induced relaxation was observed, and the presence or absence of PVAT had no effect on ACh‐induced relaxation between the groups (Figure 5e). AngII‐mediated contraction was not different between the female offspring groups, and the presence or absence of PVAT had no effect on AngII‐induced contraction. (Figure 5f).

Secondary linear mixed‐effects modeling accounting for dam as a random effect revealed no significant main or interaction effects of maternal diet, offspring diet, or sex on NE‐induced contraction in the presence or absence of PVAT, AngII‐induced contraction in the presence or absence of PVAT, or ACh‐induced relaxation in the absence of PVAT (all *p* > 0.15). For ACh‐induced relaxation in the presence of PVAT, linear mixed‐effects modeling accounting for dam as a random effect revealed no significant main or interaction effects of maternal diet or offspring diet, while there was a trend for a sex effect (female > male; estimate = 0.16380%, *p* = 0.0882).

## 4 DISCUSSION

Presently, ~60% of pregnant women are overweight, with a third being obese in the US (Catalano & Ehrenberg, 2006; Flegal et al., 2012). Thus, a high proportion of infants born in the US will have encountered an overweight/obese intrauterine environment throughout gestation. Studies have shown a positive association between maternal obesity and offspring development of obesity and other comorbidities. (Chang et al., 2019; Gambineri et al., 2020; Howie et al., 2013; Segovia et al., 2014; Shrestha et al., 2020; Wakana et al., 2015) Interestingly, the prevalence of offspring obesity was reduced in mothers who received gastric bypass surgery prior to conception (Barisione et al., 2012; Kral et al., 2006; Whitaker et al., 1997). Though these observations provide much needed insight into the outcomes of maternal obesity during pregnancy, there have been limited investigations to determine if offspring diets can attenuate maternal diet programming effects or if there are offspring sex differences in maternal programming. The work presented here aimed to address these current gaps in knowledge.

In our experimental paradigm, we observed that in general, offspring postweaning diet was the primary driver of offspring body composition, metabolism, and vascular reactivity, and that a maternal diet high in fat and sucrose prior to and during pregnancy lactation had little impact on these physiological variables. For all measured variables, we observed that offspring postweaning diet and not maternal diets were stronger predictors in both the male and female offspring. While initial ANOVA analysis showed some notable exceptions as postweaning HFD‐fed female offspring from HFD‐fed dams had significantly higher fat mass, lower lean mass, and elevated serum leptin relative to all other female diet groups, statistically, these findings did not hold up to linear mixed‐effects modeling including both sexes and accounting for dam as a random effect. Of note, leptin is a hormone produced by adipocytes that signals to the brain to regulate appetite, adiposity, and whole body metabolism, and in people and experimental animal models, serum leptin levels strongly positively correlate with body weight and body fat percentage (Ahima, 2008; Considine et al., 1996; Hamilton et al., 1995; Lönnqvist et al., 1995; Obradovic et al., 2021). The elevation in serum leptin corresponded with increased fat mass and reduction of lean mass in HH females. Though these data were not significant when accounting for sex or dam as random effects, they suggest that when female offspring from HFD‐fed mothers are similarly challenged with a postweaning HFD, maternal diet may program maladaptive offspring body composition and associated circulating adipokines. Interestingly, in male offspring, a programming trend on body composition was also noted, but this effect corresponded to less lean mass in HH offspring. In males, the combined interventions of maternal and offspring feeding of a control diet led to significantly higher lean mass percentages for the CC group relative to all others. Additionally, in male offspring, the combination of maternal and offspring postweaning HFD led to elevated offspring serum insulin concentrations. Similar to leptin, serum insulin levels are enmeshed with body composition, with excessive body mass accumulation contributing to perturbations in insulin and glucose regulation (Kahn et al., 2006; Kahn & Flier, 2000; Ye, 2013). This suggests a nuanced influence of maternal programming in our experimental paradigm as the impact of maternal diet was limited to specific conditions and diverges in effect between males (lean mass/insulin) and females (fat mass/leptin). Other groups have observed different patterns in maternal diet contribution to offspring body weight and body composition glucose handling. For example, Desai et al. observed that maternal HFD programmed hyperphagia and obesity in male offspring fed a control diet for 12 months via neurogenic mechanisms and nutrient sensor AMPK (Desai et al., 2020). Xu et al. observed that maternal HFD consumption only during lactation programmed excessive weight and adiposity in male but not female offspring when they were fed a HFD starting at 12 weeks of age (Xu et al., 2023). In a study by Yokomizo et al., dams were fed either a control or HFD from conception through weaning and then male and female offspring were fed either a control or HFD from 6 to 20 weeks of age. They observed that there was a positive maternal HFD programming effect on the body weight of female offspring and a maternal HFD programming effect on glucose intolerance and insulin resistance, with sex‐specific changes in pancreatic β‐cell function in male offspring (Yokomizo et al., 2014). An older meta‐analysis that included 171 studies looking at maternal programming concluded that maternal HFD had no effect on birthweight but was positively associated with increased offspring weight at weaning and later on in life, increased adiposity, and increased triglycerides, cholesterol, and insulin, while hyperglycemia was mainly seen in female offspring (Ribaroff et al., 2017). In summary, there are variable offspring responses to maternal HFD consumption during developmental critical periods such as pregnancy and lactation. The lack of reproducibility in these rodent maternal programming studies is multi‐factorial, including but not limited to differences in length and timing of maternal and offspring diet challenge, diet fat source and content, species studied, and timing of when offspring outcomes are assessed.

## 5 LIMITATIONS

The work presented here is not without any limitations. First, our maternal diet intervention spans several developmental critical periods, from preconception, conception, pregnancy, and lactation. Our rationale for our experimental design to span maternal high fat diet consumption throughout all these periods was to more closely mimic the human condition. While some people are successful, in the long term, changing dietary patterns and lifestyle habits to lose weight can prove challenging for many (Deslippe et al., 2023; Diabetes Prevention Program Research, G, 2009; Look, 2014), thus we chose to maintain our dams on the same diets before and during pregnancy and lactation. In rodents, preconception diet‐induced obesity was associated with increased expression of pro‐inflammatory genes and decreased expression of antioxidant genes in peri‐implantation blastocysts (Shankar et al., 2011). Likewise, in women undergoing fertility treatment, oocytes from women with overweight/obesity had increased expression of proinflammatory and oxidative stress‐related genes compared to oocytes from women who were normal weight (Ruebel et al., 2017). Maternal high fat diet feeding exclusively during lactation has also been demonstrated to program weight gain and increased adiposity in male offspring (Xu et al., 2023), providing evidence that each developmental critical period‐preconception, conception, pregnancy, and lactation–can play important roles in maternal programming. Moreover, while dams were maintained on dietary interventions throughout pregnancy and lactation, maternal body mass/composition and serum markers of metabolic status were not measured during pregnancy and lactation. Since C57BL/6J dams are notoriously sensitive to noise, odor, and husbandry changes, we opted to minimize disturbances to the dams during these periods to ensure pup survival. Another limitation of our study is that we cannot separate the programming effects of maternal body composition changes with diet from the maternal HFD consumption per se. One final limitation of our study may lie in the composition of control diet fed to offspring. Purified rodent diets, like the AIN‐76 and then AIN‐93 diets, were initially developed to reduce variability in cereal‐based diets with the intention of improving reproducibility between experiments and laboratories (Reeves et al., 1993). The AIN‐93G (G, Growth) diet has a higher fat content than the AIN‐93M (M, Maintenance) diet to support rapid growth during adolescence and to support females during reproduction and lactation (Reeves et al., 1993). We opted to maintain our offspring on a modified AIN‐93G diet (corn oil substituted for soybean oil) for the 14‐week postweaning feeding period for a simpler experimental design consisting of only 2 diets. It is possible that the higher fat content in our control diet (17% calories from fat), compared to the 10% calories from fat in the AIN‐93 M diet may have contributed to the lack of differences in arterial contractility between control and HFD fed mice.

## 6 CONCLUSION

In conclusion, in the maternal HFD feeding paradigm used in the study described here, offspring postweaning HFD had a much more pronounced impact than maternal HFD on offspring body composition and metabolic profile, and neither maternal nor offspring diet had significant effects on vascular reactivity. These results hold true for both male and female offspring, with the few instances of a maternal HFD programming effect limited to conditions wherein offspring were also fed a postweaning HFD. As such, in our study, the predominant factor influencing offspring body composition and metabolic characteristics was the offspring postweaning diet. Thus, while the maternal environment during critical developmental periods is important for disease risk later in life, our data emphasize that dietary patterns during childhood and adolescence play a critical role for determining health outcomes into adulthood and suggest that further studies are needed to fully understand the crosstalk between maternal programming and offspring dietary and lifestyle habits on health outcomes.

## AUTHOR CONTRIBUTIONS

**Adam Corken:** Data curation; formal analysis; investigation; methodology; validation; visualization. **Elizabeth C. Wahl:** Data curation; formal analysis; investigation; methodology. **James D. Sikes:** Data curation; investigation; methodology; project administration; resources. **Keshari M. Thakali:** Conceptualization; data curation; formal analysis; funding acquisition; investigation; methodology; project administration; resources; software; supervision; validation; visualization.

## FUNDING INFORMATION

This research was supported by funding from USDA ARS 6026‐10700‐001‐000D.

## CONFLICT OF INTEREST STATEMENT

The authors declare no conflicts of interest.

## ETHICS STATEMENT

The animal study protocol was approved by the University of Arkansas for Medical Sciences Institutional Animal Care and Use Committee (IPROTO202400000124, approved 04/19/2022).

## Supporting information


**Figure S1.** Body mass measurements and litter size for birth mothers. (A) Average dam body mass at the beginning of control or high fat diet administration through 12 weeks. *p < 0.001; **p < 0.0001. (B) Average changes in dam body mass relative to baseline measurements. *p < 0.05; **p(value) < 0.0001. *N* = 40 (Control); *N* = 39 (High Fat). (C) Average fat and lean mass content as a percentage of total body mass at baseline (4 weeks old) and following 12 weeks of dietary intervention. *p* < 0.0001 (Fat mass: Baseline vs. Control, Baseline vs. High Fat, Control vs. High Fat; Lean mass: Baseline vs. Control, Baseline vs. High Fat). *N* = 69 (Baseline); *N* = 40 (Control); *N* = 59 (High Fat). (D) Average percent changes in fat and lean mass relative to baseline. *p < 0.0001 (Fat mass: Control vs. High Fat). *N* = 20 (Control); *N* = 27 (HFD). (E) Average litter size for each dam cohort, *p* = 0.0076 (Control vs. High Fat). *N* = 40 (Control); *N* = 59 (High Fat).
**Figure S2:** Body composition measurements. (A) Male fat mass at baseline, 7 and 14 weeks of feeding. *p < 0.0001 [CC v CH]; **p < 0.0001 [CC v HH]; ***p < 0.0001 [HC vs. CH]; ^X^ *p* <0.0001 [HC vs. HH]; ^XX^ *p* = 0.0049 [CH vs. HH]; ^XXX^ *p* = 0.0004 [CH v HH]. (B) Male lean mass at baseline, 7 and 14 weeks of dietary intervention. *p < 0.0001 [CC v HH]; **p = 0.0061 [HC vs. HH]; ***p = 0.0159 [CH vs. HH]; ^X^ *p*(value) = 0.0045 [CC vs. HH]; ^XX^ *p* = 0.03 [HC vs. HH]. (C) Change in fat and lean mass in male offspring from baseline following 14 weeks of diet. *p* < 0.0001 (Fat mass: CC vs. HH, CC vs. CH, HC vs. HH, HC vs. CH, CH vs. HH; Lean mass: HC vs. HH), *p* = 0.0063 (Lean mass: CC vs. HH), *p* = 0.0373 (Lean mass: HC vs. CH). (D) Female offspring fat mass at baseline, 7 and 14 weeks of feeding. *p < 0.0001 [CC vs. CH]; **p < 0.0001 [CC vs. HH]; ***p < 0.0001 [HC vs. HH]; ^X^ *p* <0.0001 [CH vs. HH]; ^XX^ *p* = 0.0028 [CC vs. HC]; ^XXX^ *p* = 0.0009 [CC vs. HC]. (E) Female lean mass at baseline, 7 and 14 weeks of diet. *p < 0.0001 [CC vs. HH]; **p < 0.0001 [CC vs. CH]; ***p < 0.0001 [HC vs. HH]; ^**X**^ *p* = 0.0011 [CH vs. CH]; ^**XX**^ *p* = 0.0385 [HC vs. HH]. (F) Female offspring fat and lean mass changes from baseline to 14 weeks of diet. *p* < 0.0001 (Fat mass: CC vs. HH, CC vs. CH, HC vs. HH, HC vs. CH, CH vs. HH; Lean mass: CC vs. CH), *p* = 0.0451 (Fat mass: CC vs. HC), *p* = 0.0003 (Lean mass: CC vs. HH), *p* = 0.0253 (Lean mass: HC vs. HH), *p* = 0.0168 (Lean Mass: HC vs. CH). Males, *N* = 42 (CC); *N* = 32 (HC); *N* = 46 (CH); *N* = 36 (HH). Females, *N* = 56 (CC); *N* = 28 (HC); *N* = 57 (CH); *N* = 30 (HH).
**Figure S3:** Indirect calorimetry metabolic measurements of male and female offspring. (A) Cumulative food intake over 48 h in male offspring. (B) Cumulative food intake over 48 h in female offspring. *p* = 0.0282 (Dark: CC vs. CH), *p* < 0.0001 (Dark: HC vs. CH), *p* = 0.0117 (Light: CC vs. CH), *p* = 0.0222 (Light HC vs. CH). Males, *N* = 27 (CC); *N* = 27 (HC); *N* = 24 (CH); *N* = 28 (HH). Females, *N* = 39 (CC); *N* = 24 (HC); *N* = 39 (CH); *N* = 27 (HH). Box plots depict the lower and upper quartile of the data sets as the outer borders with median value in between. The whiskers represent the minimum and maximum values of the data set. (HH).

## Data Availability

The dataset outlined in this manuscript is available from the corresponding author upon request.
