# Supplementary material for: Postweaning diet affects offspring metabolic outcomes more than maternal diet in male and female mice
Source: Physiol Rep. 2026 Apr 20;14(8):e70881. doi: 10.14814/phy2.70881 (PMC13095646; doi:10.14814/phy2.70881)
Supplement: Supplementary file 1 — Figure S1. Body mass measurements and litter size for birth mothers. (A) Average dam body mass at the beginning of control or high fat diet administration through 12 weeks. *p < 0.001; **p < 0.0001. (B) Average changes in dam body mass relative to baseline measurements. *p < 0.05; **p(value) < 0.0001. N = 40 (Control); N = 39 (High Fat). (C) Average fat and lean mass content as a percentage of total body mass at baseline (4 weeks old) and following 12 weeks of dietary intervention. p < 0.0001 (Fat mass: Baseline vs. Control, Baseline vs. High Fat, Control vs. High Fat; Lean mass: Baseline vs. Control, Baseline vs. High Fat). N = 69 (Baseline); N = 40 (Control); N = 59 (High Fat). (D) Average percent changes in fat and lean mass relative to baseline. *p < 0.0001 (Fat mass: Control vs. High Fat). N = 20 (Control); N = 27 (HFD). (E) Average litter size for each dam cohort, p = 0.0076 (Control vs. High Fat). N = 40 (Control); N = 59 (High Fat). Figure S2: Body composition measurements. (A) Male fat mass at baseline, 7 and 14 weeks of feeding. *p < 0.0001 [CC v CH]; **p < 0.0001 [CC v HH]; ***p < 0.0001 [HC vs. CH]; X p <0.0001 [HC vs. HH]; XX p = 0.0049 [CH vs. HH]; XXX p = 0.0004 [CH v HH]. (B) Male lean mass at baseline, 7 and 14 weeks of dietary intervention. *p < 0.0001 [CC v HH]; **p = 0.0061 [HC vs. HH]; ***p = 0.0159 [CH vs. HH]; X p(value) = 0.0045 [CC vs. HH]; XX p = 0.03 [HC vs. HH]. (C) Change in fat and lean mass in male offspring from baseline following 14 weeks of diet. p < 0.0001 (Fat mass: CC vs. HH, CC vs. CH, HC vs. HH, HC vs. CH, CH vs. HH; Lean mass: HC vs. HH), p = 0.0063 (Lean mass: CC vs. HH), p = 0.0373 (Lean mass: HC vs. CH). (D) Female offspring fat mass at baseline, 7 and 14 weeks of feeding. *p < 0.0001 [CC vs. CH]; **p < 0.0001 [CC vs. HH]; ***p < 0.0001 [HC vs. HH]; X p <0.0001 [CH vs. HH]; XX p = 0.0028 [CC vs. HC]; XXX p = 0.0009 [CC vs. HC]. (E) Female lean mass at baseline, 7 and 14 weeks of diet. *p < 0.0001 [CC vs. HH]; **p < 0.0001 [ [file PHY2-14-e70881-s001.docx]

Supplementary Material

Postweaning Diet affects Offspring Metabolic Outcomes More than Maternal Diet in Male and Female Mice

Adam Corken^1,2^, Elizabeth C. Wahl^2^, James D. Sikes^2^, and Keshari M. Thakali^1,2^*

^1^Department of Pediatrics, University of Arkansas for Medical Sciences, Little Rock, AR, USA; e-mail@e-mail.com

^2^Arkansas Children’s Research Institute, Little Rock, AR, USA


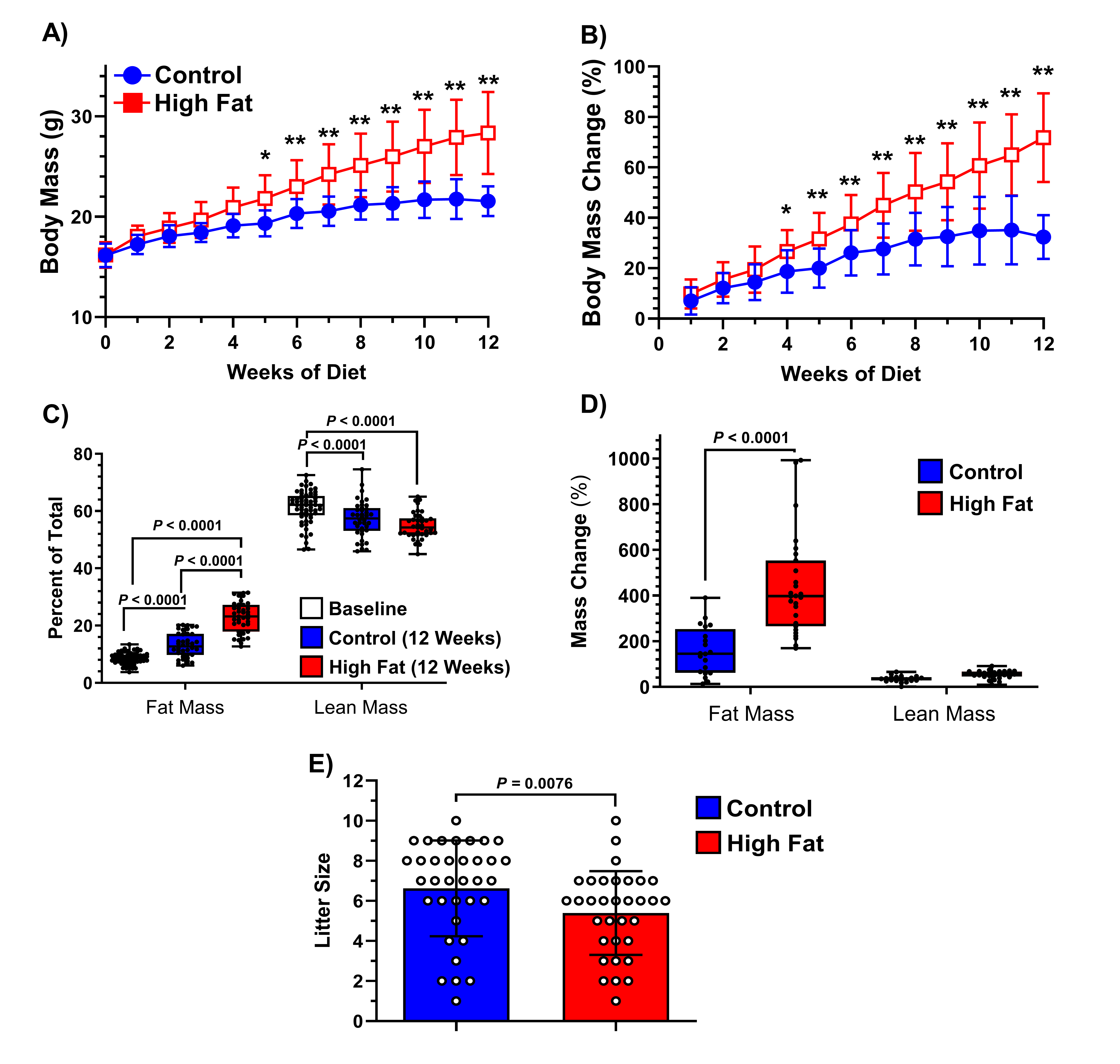
*****Correspondence: KMThakali@uams.edu

**Figure 1: Body Mass Measurements and Litter Size for Birth Mothers. A)** Average dam body mass at the beginning of control or high fat diet administration through 12 weeks. *P < 0.001; **P < 0.0001. **B)** Average changes in dam body mass relative to baseline measurements. *P < 0.05; **P(value) < 0.0001. N = 40 (Control); N = 39 (High Fat). **C)** Average fat and lean mass content as a percentage of total body mass at baseline (4 weeks old) and following 12 weeks of dietary intervention. P < 0.0001 (Fat mass: Baseline vs Control, Baseline vs High Fat, Control vs High Fat; Lean mass: Baseline vs Control, Baseline vs High Fat). N = 69 (Baseline); N = 40 (Control); N = 59 (High Fat). **D)** Average percent changes in fat and lean mass relative to baseline. *P < 0.0001 (Fat mass: Control vs High Fat). N = 20 (Control); N = 27 (HFD). **E)** Average litter size for each dam cohort, P = 0.0076 (Control vs High Fat). N = 40 (Control); N = 59 (High Fat).


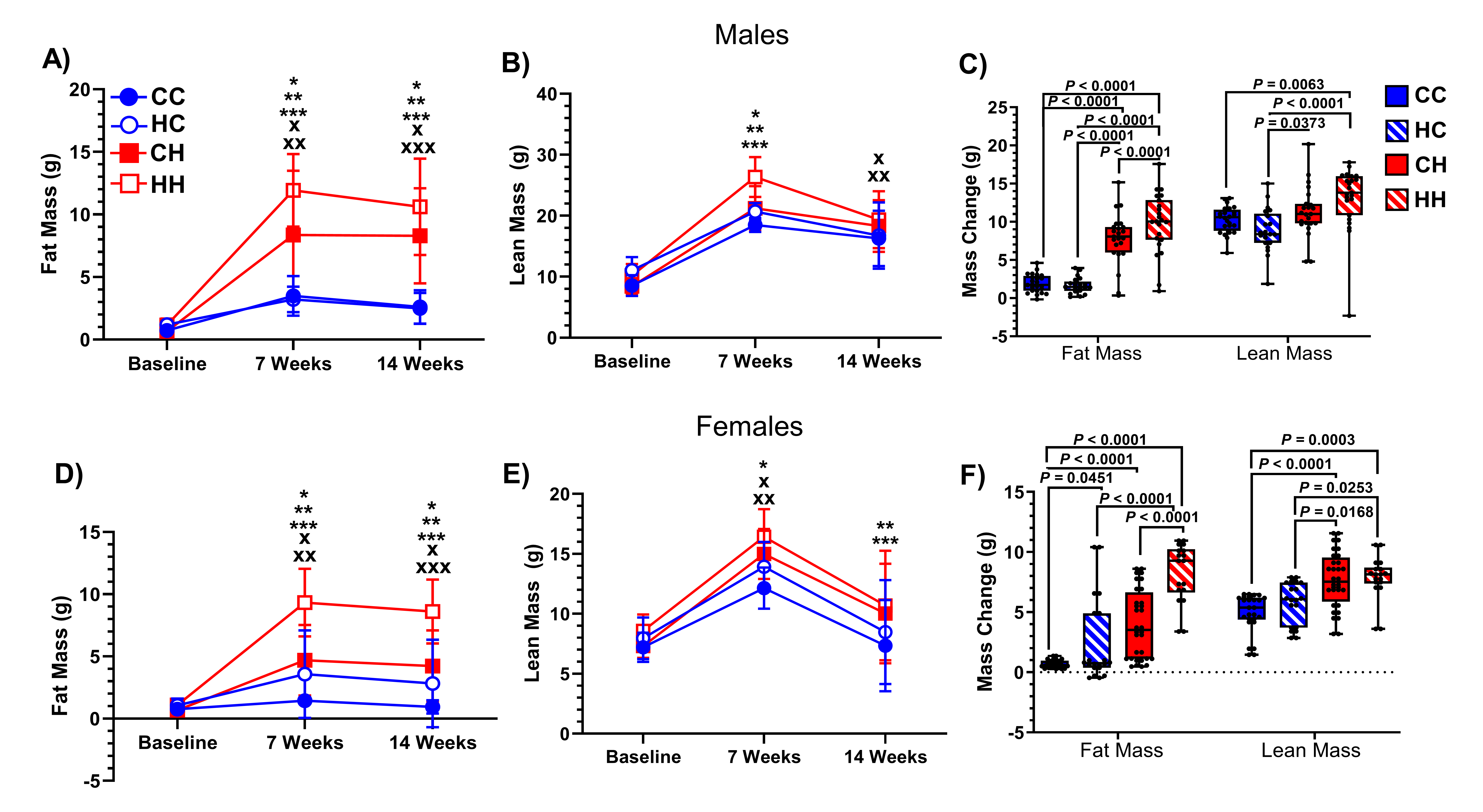
**Figure 2: Body Composition Measurements. A)** Male fat mass at baseline, 7 and 14 weeks of feeding. *P < 0.0001 [CC v CH]; **P < 0.0001 [CC v HH]; ***P < 0.0001 [HC v CH]; **^X^**P < 0.0001 [HC v HH]; **^XX^**P = 0.0049 [CH v HH]; **^XXX^**P = 0.0004 [CH v HH]. **B)** Male lean mass at baseline, 7 and 14 weeks of dietary intervention. *P < 0.0001 [CC v HH]; **P = 0.0061 [HC v HH]; ***P = 0.0159 [CH v HH]; **^X^**P(value) = 0.0045 [CC v HH]; **^XX^**P = 0.03 [HC v HH]. **C)** Change in fat and lean mass in male offspring from baseline following 14 weeks of diet. P < 0.0001 (Fat mass: CC vs HH, CC vs CH, HC vs HH, HC vs CH, CH vs HH; Lean mass: HC vs HH), P = 0.0063 (Lean mass: CC vs HH), P = 0.0373 (Lean mass: HC vs CH). **D)** Female offspring fat mass at baseline, 7 and 14 weeks of feeding. *P < 0.0001 [CC v CH]; **P < 0.0001 [CC v HH]; ***P < 0.0001 [HC v HH]; **^X^**P < 0.0001 [CH v HH]; **^XX^**P = 0.0028 [CC v HC]; **^XXX^**P = 0.0009 [CC v HC]. **E)** Female lean mass at baseline, 7 and 14 weeks of diet. *P < 0.0001 [CC v HH]; **P < 0.0001 [CC v CH]; ***P < 0.0001 [HC v HH]; **^X^**P = 0.0011 [CH v CH]; **^XX^**P = 0.0385 [HC v HH]. **F)** Female offspring fat and lean mass changes from baseline to 14 weeks of diet. P < 0.0001 (Fat mass: CC vs HH, CC vs CH, HC vs HH, HC vs CH, CH vs HH; Lean mass: CC vs CH), P = 0.0451 (Fat mass: CC vs HC), P = 0.0003 (Lean mass: CC vs HH), P = 0.0253 (Lean mass: HC vs HH), P = 0.0168 (Lean Mass: HC vs CH). Males, N = 42 (CC); N = 32 (HC); N = 46 (CH); N= 36 (HH). Females, N = 56 (CC); N = 28 (HC); N = 57 (CH); N = 30 (HH).

**Figure 3: Indirect calorimetry metabolic measurements of male and female offspring. A)** Cumulative food intake over 48 hours in male offspring. **B)** Cumulative food intake over 48 hours in female offspring. P = 0.0282 (Dark: CC vs CH), P <0.0001 (Dark: HC vs CH), P = 0.0117 (Light: CC vs CH), P = 0.0222 (Light HC vs CH). Males, N = 27 (CC); N = 27 (HC); N = 24 (CH); N = 28 (HH). Females, N = 39 (CC); N = 24 (HC); N = 39 (CH); N = 27 (HH). Box plots depict the lower and upper quartile of the data sets as the outer borders with median value in between. The whiskers represent the minimum and maximum values of the data set. (HH).
